# Perinatal High-Fat Diet Influences Ozone-Induced Responses on Pulmonary Oxidant Status and the Molecular Control of Mitophagy in Female Rat Offspring

**DOI:** 10.3390/ijms22147551

**Published:** 2021-07-14

**Authors:** Sven H. Rouschop, Samantha J. Snow, Urmila P. Kodavanti, Marie-José Drittij, Lou M. Maas, Antoon Opperhuizen, Frederik J. van Schooten, Alexander H. Remels, Roger W. Godschalk

**Affiliations:** 1 Department of Pharmacology and Toxicology, NUTRIM School of Nutrition and Translational Research in Metabolism, Maastricht University, 6200 Maastricht, The Netherlands; s.rouschop@maastrichtuniversity.nl (S.H.R.); mj.drittij@maastrichtuniversity.nl (M.-J.D.); l.maas@maastrichtuniversity.nl (L.M.M.); antoon.opperhuizen@maastrichtuniversity.nl (A.O.); f.vanschooten@maastrichtuniversity.nl (F.J.v.S.); a.remels@maastrichtuniversity.nl (A.H.R.); 2 Public Health and Integrated Toxicology Division, Center for Public Health and Environmental Assessment, Office of Research and Development, U.S. Environmental Protection Agency, Durham, NC 27711, USA; samanthajsnow@gmail.com (S.J.S.); Kodavanti.urmila@epa.gov (U.P.K.); 3 ICF International Inc., Durham, NC 27711, USA; 4 Netherlands Food and Consumer Product Safety Authority (NVWA), 3511 Utrecht, The Netherlands

**Keywords:** high-fat diet, obesity, perinatal, ozone, pulmonary toxicity, mitochondria, oxidative stress

## Abstract

Previous research has shown that a perinatal obesogenic, high-fat diet (HFD) is able to exacerbate ozone-induced adverse effects on lung function, injury, and inflammation in offspring, and it has been suggested that mitochondrial dysfunction is implicated herein. The aim of this study was to investigate whether a perinatal obesogenic HFD affects ozone-induced changes in offspring pulmonary oxidant status and the molecular control of mitochondrial function. For this purpose, female Long-Evans rats were fed a control diet or HFD before and during gestation, and during lactation, after which the offspring were acutely exposed to filtered air or ozone at a young-adult age (forty days). Directly following this exposure, the offspring lungs were examined for markers related to oxidative stress; oxidative phosphorylation; and mitochondrial fusion, fission, biogenesis, and mitophagy. Acute ozone exposure significantly increased pulmonary oxidant status and upregulated the molecular machinery that controls receptor-mediated mitophagy. In female offspring, a perinatal HFD exacerbated these responses, whereas in male offspring, responses were similar for both diet groups. The expression of the genes and proteins involved in oxidative phosphorylation and mitochondrial biogenesis, fusion, and fission was not affected by ozone exposure or perinatal HFD. These findings suggest that a perinatal HFD influences ozone-induced responses on pulmonary oxidant status and the molecular control of mitophagy in female rat offspring.

## 1. Introduction

The prevalence of obesity has nearly doubled since 1980 [1], resulting in 52% of the European Union’s (EU) adult population being overweight and 16% being obese [2]. Maternal obesity during pregnancy negatively affects the offspring’s health, predisposing the offspring to obesity, type 2 diabetes, and cardiovascular diseases [3,4,5,6]. Furthermore, prenatal obesity affects lung health, with children from obese mothers being more susceptible to wheezing and developing asthma [7,8]. Similarly, a prenatal obesogenic high-fat diet (HFD) in rats and mice has been shown to induce airway hyperresponsiveness, airway inflammation, and increased susceptibility to respiratory infections [9,10]. Because of these adverse pulmonary effects, the offspring from obese mothers may be more susceptible to environmental exposures affecting the health of the lung, such as ozone.

Ozone is a secondary air pollutant that originates from photochemical reactions between precursor pollutants, such as nitrogen oxides (NO_x_) and volatile organic compounds (VOCs), under the influence of sunlight. Upon inhalation, ozone has various adverse effects on the lungs, such as decreased lung function, increased lung inflammation, airway hyperresponsiveness, and increased pulmonary vascular permeability [11,12,13,14,15]. To protect the population against these pulmonary responses and other harmful effects induced by ozone, a target value of 0.06 ppm was set by the EU for air quality standards. Nevertheless, in 2018, 34% of the EU’s urban population was exposed to ozone concentrations sufficiently high to cause harmful pulmonary effects [16].

Despite the increasing obesity prevalence and a large part of the urban population being exposed to harmful ozone levels, little research has been done on the combined effects of prenatal obesity and air pollution on the lungs of offspring. So far, two studies have shown that ozone-induced detrimental effects on lung function, injury, and inflammation in rat offspring were more profound in the offspring from dams that were fed a HFD diet during gestation [17,18]. Furthermore, a study deploying a systemic metabolomics approach demonstrated that a perinatal HFD exacerbated ozone-induced effects on lipid, protein, and energy metabolism, which suggested an impairment of mitochondrial function [19]. To elucidate whether mitochondria and changes in the molecular machinery that control the mitochondrial content and function are involved in the effect of maternal obesity on ozone susceptibility in offspring, we performed an experiment in which female rats were fed a HFD before and during gestation, and during lactation, after which the offspring were exposed to ozone at a young-adult age (forty days). Directly following this exposure, the offspring lungs were examined for markers related to mitochondrial content and function, such as oxidative stress; oxidative phosphorylation; and mitochondrial fusion, fission, biogenesis, and mitophagy. In this paper, we show that acute ozone exposure increases oxidative status and upregulates the molecular control of receptor-mediated mitophagy in the lungs of young-adult rats, and that these responses are exacerbated in female offspring from dams fed a perinatal HFD.

## 2. Results

### 2.1. Acute Ozone Exposure Increases Pulmonary Oxidant Status

**Figure 1:**
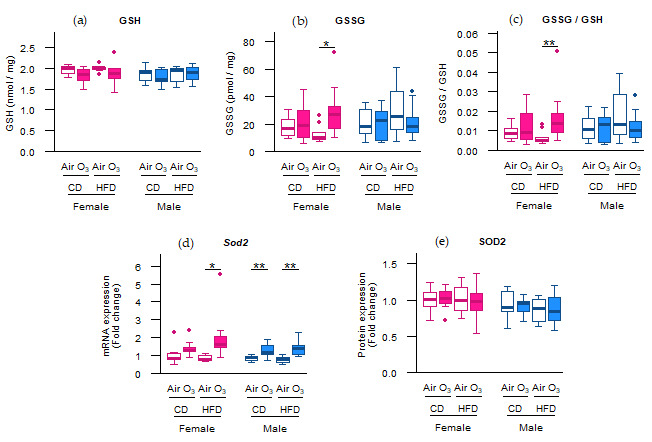
Acute ozone exposure increased pulmonary oxidant status in female offspring from high-fat diet (HFD) dams and all male offspring. (**a**) Reduced glutathione (GSH), (**b**) oxidized glutathione (GSSG) and (**c**) GSSG/GSH ratio, (**d**) *Sod2* mRNA expression, and (**e**) SOD2 protein expression were assessed within two hours after exposure to filtered air or ozone in the lungs of female and male offspring from dams fed a perinatal control diet (CD) or HFD. Data are presented as boxplots. Statistical analysis by two-way ANOVA with a Holm–Sidak post-hoc test. *n* = 10 per group (**a**–**c**), *n* = 9–10 per group (**d**), or *n* = 7–10 per group (**e**). * *p* < 0.05, ** *p* < 0.01. O_3_—ozone.

We previously showed that the perinatal HFD compared with the CD increased the body weight and fat percentage of dams throughout gestation, as well as of the offspring at postnatal day (PND) 37 [18,19]. Furthermore, acute ozone exposure was shown to cause pulmonary protein leakage and lung cell injury in both female and male offspring, and induced neutrophilic inflammation in female offspring [19]. We next studied whether these ozone-induced pulmonary responses in offspring were associated with alterations in oxidative stress markers and whether these responses were affected by maternal diet. For the antioxidant glutathione, reduced (GSH) levels were not affected (Figure 1a), but ozone exposure did significantly increase the levels of oxidized glutathione (GSSG), and therefore the GSSG/GSH ratio, compared with air exposure in female offspring from HFD dams (Figure 1b,c). Ozone exposure also significantly upregulated mRNA expression of the mitochondrial antioxidant superoxide dismutase 2 (*Sod2*) in female offspring from HFD dams, and male offspring from both CD and HFD dams (Figure 1d). In addition, *Sod2* mRNA levels correlated significantly with previously published [19] pulmonary vascular leakage markers albumin and total protein in broncheoalveolar lavage fluid (BALF) in female and male offspring from HFD dams (Table 1). Altogether, these data suggest that acute ozone exposure increased the pulmonary oxidant status in female offspring from HFD dams and all male offspring.

**Figure 2:**
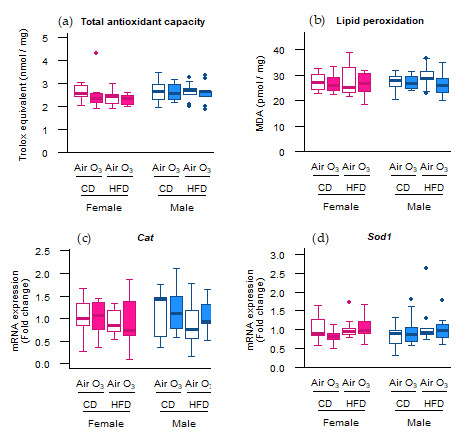
Acute ozone exposure and perinatal high-fat diet (HFD) did not alter the pulmonary (**a**) total antioxidant capacity, (**b**) lipid peroxidation, or expression of cytoplasmic antioxidant genes (**c**) *Cat* and (**d**) *Sod1*. All of the markers were assessed within two hours after exposure to filtered air or ozone in the lungs of female and male offspring from dams fed a perinatal control diet (CD) or HFD. Data are presented as boxplots. Statistical analysis by two-way ANOVA with a Holm–Sidak post-hoc test. *n* = 10 per group (**a**,**b**) or *n* = 9–10 group (**c**,**d**). O_3_—ozone.

Not all oxidative stress markers, however, were affected by ozone exposure. Although the *Sod2* mRNA levels were upregulated in most offspring groups, this did not lead to increased SOD2 protein levels in any of the offspring groups (Figure 1e). Furthermore, no differences were observed in the Trolox equivalent antioxidant capacity (TEAC) and levels of the lipid peroxidation product malondialdehyde (MDA) (Figure 2a,b), indicating that acute ozone exposure and perinatal HFD did not alter the total antioxidant capacity or induce oxidative damage to lipids, respectively, in the offspring lungs. The mRNA expression of cytoplasmic antioxidants catalase (*Cat*) and superoxide dismutase 1 (*Sod1*) were also not altered by ozone exposure or perinatal HFD (Figure 2c,d).

### 2.2. Acute Ozone Exposure Alters the Abundance of Key Constituents Involved in Receptor-Mediated Mitophagy

**Figure 3:**
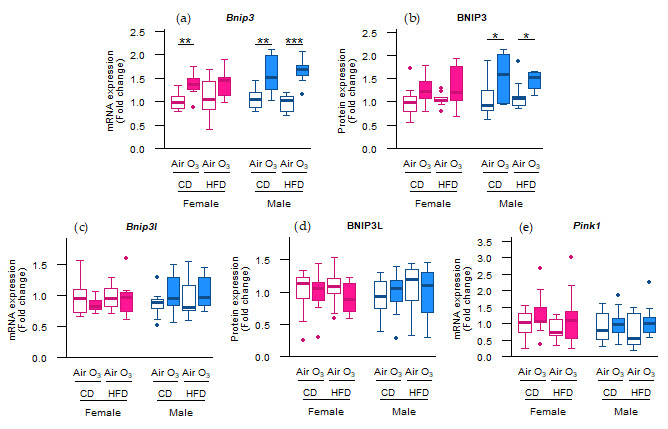
Acute ozone exposure altered the molecular control of receptor-mediated mitophagy rather than ubiquitin-mediated mitophagy. Receptor-mediated mitophagy: (**a**) *Bnip3* mRNA expression, (**b**) BNIP3 protein expression, (**c**) *Bnip3l* mRNA expression, (**d**) and BNIP3L protein expression. Ubiquitin-mediated mitophagy: (**e**) *Pink1* mRNA expression. All of the markers were assessed within two hours after exposure to filtered air or ozone in lungs of female and male offspring from dams fed a perinatal control diet (CD) or high-fat diet (HFD). Data are presented as boxplots. Statistical analysis by two-way ANOVA with a Holm–Sidak post-hoc test. *n* = 9–10 per group (**a**,**c**,**e**) or *n* = 7–10 per group (**b**,**d**). * *p* < 0.05, ** *p* < 0.01, *** *p* < 0.001. O_3_—ozone.

As acute ozone exposure upregulated the mRNA expression of the mitochondrial antioxidant *Sod2*, but not of the cytoplasmic antioxidants *Cat* and *Sod1*, we next examined whether the molecular control of the mitochondrial content in the offspring lungs was affected. Considering that mitochondria are not only a major source of reactive oxygen species (ROS), but can also be damaged by it, we first assessed the gene and protein expression of key regulators of mitophagy. As a result, the mRNA expression of B-cell lymphoma 2/adenovirus E1B 19 kDa protein-interacting protein 3 (*Bnip3*), a mitophagy receptor [20], was significantly upregulated after ozone exposure in female offspring from CD dams and all male offspring (Figure 3a). Similarly, the BNIP3 protein expression was significantly increased after ozone exposure in the male offspring from CD and HFD dams (Figure 3b). The mRNA and protein expression of B-cell lymphoma 2/adenovirus E1B 19 kDa protein-interacting protein 3-like (Bnip3l), a mitophagy receptor with a close homology to Bnip3 [21], were not affected by ozone exposure or perinatal HFD (Figure 3c,d). Likewise, no differences were observed in the mRNA expression of phosphatase and tensin homolog-induced kinase 1 (*Pink1*; Figure 3e), a gene essential for ubiquitin-mediated mitophagy [22]. These results suggest that acute ozone exposure induced changes in the abundance of the molecular machinery controlling the receptor-mediated mitophagy in the offspring lungs rather than ubiquitin-mediated mitophagy in the female offspring from CD dams and all male offspring.

### 2.3. Acute Ozone Exposure and Perinatal HFD Do Not Alter the Expression of the Oxidative Phosphorylation Machinery

**Figure 4:**
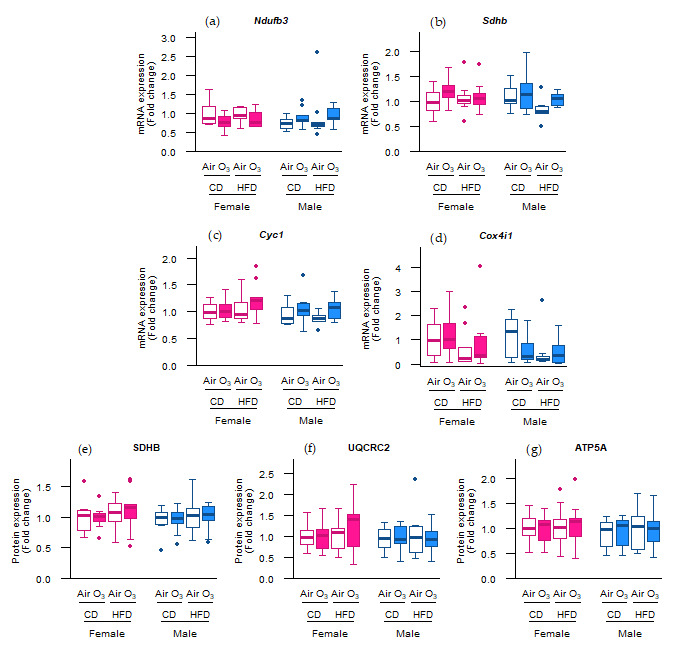
Perinatal high-fat diet (HFD) or acute ozone exposure did not change the pulmonary expression of oxidative phosphorylation machinery subunits. mRNA expression of (**a**) *Ndufb3* (complex I), (**b**) *Sdhb* (complex II), (**c**) *Cyc1* (complex III), and (**d**) *Cox4i1* (complex IV), and the protein expression of (**e**) SDHB (complex II), (**f**) UQCR2 (complex III), and (**g**) ATP5A (complex V) were assessed within two hours after exposure to filtered air or ozone in the lungs of female and male offspring from dams fed a perinatal control diet (CD) or HFD. Data are presented as boxplots. Statistical analysis by two-way ANOVA with Holm–Sidak post-hoc test. *n* = 9–10 per group (**a**–**d**) or *n* = 7–10 per group (**e**–**g**). O_3_—ozone.

As ozone exposure seemed to alter the molecular regulation of mitophagy, we further assessed the abundance of key constituents of the complexes involved in oxidative phosphorylation. However, no differences were observed in the mRNA and protein levels of subunits of oxidative phosphorylation complexes I to V (Figure 4a–g), demonstrating that acute ozone exposure or perinatal HFD did not affect the expression of the oxidative phosphorylation machinery in the offspring lungs.

### 2.4. Acute Ozone Exposure and Perinatal HFD Do Not Affect the Abundance of Key Regulators of Mitochondrial Biogenesis, Fission or Fusion in Lung Tissue

To further test whether the molecular control of the mitochondrial content was affected, we measured the expression of the master regulators of mitochondrial biogenesis peroxisome proliferator-activated receptor gamma coactivator 1-alpha (*Ppargc1a*) and peroxisome proliferator-activated receptor gamma coactivator 1-beta (*Ppargc1b*), and of a key regulator of mitochondrial gene expression transcription factor a, mitochondrial (*Tfam*).

**Figure 5:**
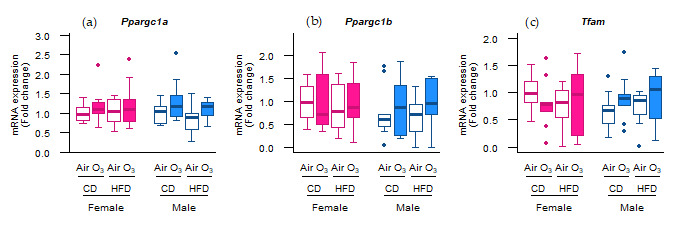
Perinatal high-fat diet (HFD) or acute ozone exposure did not affect the molecular regulation of mitochondrial biogenesis. (**a**) *Ppargc1a*, (**b**) *Ppargc1b*, and (**c**) *Tfam* mRNA levels were measured within two hours after exposure to filtered air or ozone in the lungs of female and male offspring from dams fed a perinatal control diet (CD) or HFD. Data are presented as boxplots. Statistical analysis by two-way ANOVA with a Holm–Sidak post-hoc test. *n* = 9–10 per group. O_3_—ozone.

**Figure 6:**
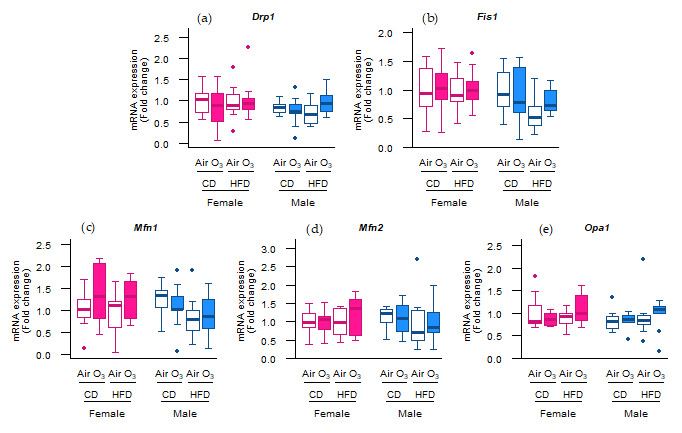
Acute ozone exposure and perinatal high-fat diet (HFD) did not alter the pulmonary molecular regulation of mitochondrial fission or fusion. Expression of fission genes (**a**) *Drp1* and (**b**) *Fis1*, and fusion genes (**c**) *Mfn1*, (**d**) *Mfn2*, and (**e**) *Opa1* was measured within two hours after exposure to filtered air or ozone in the lungs of female and male offspring from dams fed a perinatal control diet (CD) or HFD. Data are presented as boxplots. Statistical analysis by two-way ANOVA with a Holm–Sidak post-hoc test. *n* = 9–10 per group. O_3_—ozone.

However, the mRNA expression of these genes was not altered (Figure 5), indicating that mitochondrial biogenesis was not affected by acute ozone exposure or perinatal HFD. Next to the mitophagy and biogenesis, mitochondrial function and content is also regulated through the events of fission and fusion. To examine whether mitochondrial fission and fusion were altered by ozone exposure or perinatal HFD, the gene expression of fission mediators dynamin-related protein 1 (*Drp1*) and mitochondrial fission protein 1 (*Fis1*) was examined, as well as the gene expression of fusion mediators mitofusin-1 (*Mfn1*), mitofusin-2 (*Mfn2*), and optic atrophy 1 (*Opa1*). No differences were observed, however, in the mRNA levels of any of these genes (Figure 6a–e). Altogether, these data suggest that the processes of mitochondrial biogenesis, fission, and fusion were likely not affected in the offspring lungs by acute ozone exposure or perinatal HFD.

## 3. Discussion

Previous research has shown that a perinatal HFD is able to exacerbate ozone-induced pulmonary responses, and has suggested mitochondrial dysfunction to be involved in this effect [17,18,19]. Whether or not disturbances in the molecular regulation of the mitochondrial content and function are associated with this, however, is unknown. We therefore examined the lungs of offspring exposed to a perinatal HFD and ozone for molecular markers related to oxidative stress and the regulation of the mitochondrial content. We showed that acute ozone exposure increased pulmonary oxidant status and upregulated the abundance of the key constituents involved in the molecular control of receptor-mediated mitophagy. For female offspring, perinatal HFD aggravated these ozone responses, whereas for male offspring, the responses were similar in both diet groups. Abundances of mRNAs and proteins involved in oxidative phosphorylation and mitochondrial biogenesis, fusion, and fission were not affected by ozone exposure or perinatal HFD.

First, we showed that acute ozone exposure upregulated the mRNA expression of the antioxidant gene *Sod2* in all offspring, except for the females from CD dams. In addition, the glutathione oxidation status (GSSG/GSH ratio) was increased in female offspring from HFD females. Collectively, these results suggest that ozone exposure increased the pulmonary oxidant status, and that perinatal HFD aggravated these responses in female offspring only. The upregulation of Sod2 in response to both acute and chronic ozone exposure had previously been shown in other animal studies [23,24]. In addition, both Sod2 and glutathione appear to play an important role in protecting the mitochondria against ozone-induced oxidative stress. For instance, Sod2 overexpression and glutathione supplementation reduced p62 knockout-induced mitochondrial ROS in mouse embryonic fibroblasts [25]. Furthermore, increasing the intracellular GSH pool in yeast was shown to reduce fasting-induced mitophagy [26,27], but not other non-selective forms of autophagy [26]. In addition, genetic linkage analyses have linked the *Sod2* and glutathione peroxidase (*Gpx1*) genes to differential ozone susceptibility in mice [28,29]. Next to these specific effects on Sod2 and glutathione, ozone exposure has also previously been shown to induce pulmonary oxidative stress in general. In both acute and chronic exposure studies in mice, ozone exposure induced pulmonary oxidative stress [30,31,32]. The administration of MitoQ or MitoTEMPO, which are a mitochondrion-specific antioxidant and an inhibitor of mitochondrial ROS, respectively, reversed not only the ozone-induced oxidative stress, but also restored other ozone-induced pulmonary responses, such as lung inflammation and airway hyperresponsiveness [30,31,32], suggesting that oxidative stress is an important mediator of other, more phenotypical pulmonary ozone responses. This may explain the correlations we found between the *Sod2* mRNA levels and BALF albumin and total protein levels, which are both markers for lung damage.

In contrast with *Sod2* expression, acute ozone exposure and perinatal HFD did not affect the mRNA expression of *Cat* and *Sod1*. Similar to Sod2, Sod1 is responsible for converting superoxide to hydrogen peroxide and oxygen, whereas Cat catalyzes the decomposition of hydrogen peroxide into water and oxygen. Cat and Sod1 exert their functions mainly in the cytoplasm and peroxisomes, respectively, while Sod2 is located in the mitochondrial matrix. Given the different effects of ozone and perinatal HFD on expression of Sod2 vs Sod1 and Cat, and taking into account the different localizations of these enzymes, our results may suggest that the oxidative response to ozone exposure was focused at the mitochondria rather than other cellular locations.

The MDA levels were not affected by perinatal HFD or ozone exposure, indicating that neither of these treatments induced oxidative damage to lipids in the offspring lungs. The absence of lipid peroxidation could imply that cells were sufficiently protected by cellular antioxidant systems against the ozone-induced increase in oxidant status. Alternatively, oxidative damage may have occurred at a later time point after ozone exposure, as was previously observed in other studies. Xu et al., for instance, reported elevated MDA levels in BALF after acute ozone exposure in mice [32]. However this outcome was assessed 24 h after exposure, providing the oxidative damage a larger period of time to develop than the two hours used in our study. In addition, Xu et al. exposed the animals to an ozone concentration of 2.5 ppm, which is three times higher than our 0.8 ppm. In a study by Li et al., six weeks of ozone exposure did not change the MDA levels in BALF [24]. However, after another six weeks, during which ozone exposure ceased, the MDA levels were increased, indicating a delayed oxidant response. Possibly, lipid peroxidation would have been increased in our study as well, if the pulmonary MDA levels were assessed at a later time point.

In our study, acute ozone exposure increased *Bnip3* mRNA expression in all offspring except the females from HFD dams, and increased BNIP3 protein expression in all male offspring. Although many studies have examined the effects of ozone exposure on mitochondrial function and content in general, the current study is the first report, to our knowledge, on the ozone effects on specific mitophagy-related markers. The accumulation of mitophagy receptors such as BNIP3 on the mitochondrial outer membrane initiate mitophagy by promoting the binding of the mitochondrial membrane to the autophagosomal membrane [33]. In light of this, the increased abundance of Bnip3 in our study may have led to a rise in mitophagy as well. Mitophagy aids in maintaining mitochondrial quality [34] and plays an important role in protecting tissues against many forms of injury, such as alcoholic liver disease [35], ischemia/reperfusion-induced acute kidney injury [36], and tumorigenesis [37]. The upregulation of the molecular control of mitophagy in our study may thus be interpreted as a beneficial response, potentially protecting the lung cells against ozone-induced injury. Female offspring from HFD dams were the only offspring to not upregulate Bnip3 in response to ozone, thereby potentially lacking the protection that an upregulation of mitophagy would offer. This lack of protection against ozone-induced oxidative stress is indeed implied by the increased oxidative status in female offspring from HFD dams, as indicated by the elevated GSSG/GSH ratio and *Sod2* expression. These results suggest that female offspring from dams fed an HFD experienced more detrimental effects from ozone exposure than the other offspring groups. This is in accordance with our previous study [19], which found ozone-induced changes in the gut microbiome metabolites and metabolites indicative of mitochondrial stress in tissues to be more profound in these same female offspring from HFD dams than other offspring groups. For males, the ozone effects did not differ between the offspring from CD or HFD dams. Altogether, these findings suggest that a perinatal HFD influences ozone-induced responses in females, but not in males. The mechanism underlying this sex-specific effect may involve gonadal hormones, as previously suggested by the findings from an ozone exposure study using gonadectomized animals [38]. In this experiment, acute ozone exposure elicited greater detrimental effects in sham-operated females than in males. Moreover, lowering gonadal hormone levels by removing the ovaries and testes of females and males, respectively, attenuated ozone-induced effects in females, and aggravated ozone responses in males, indicating the association between sex hormones and ozone susceptibility.

Given the upregulation of the molecular control of mitophagy in our study, we hypothesized that abundance of key constituents and regulators of oxidative phosphorylation, biogenesis, fusion, and fission may also be altered. However, the expression of none of the assessed markers was affected by the perinatal HFD or acute ozone exposure. This finding is in contrast with other studies examining the effect of ozone on mitochondria. In a chronic exposure study in mice, ozone exposure downregulated the pulmonary expression of the gene pathways involved in mitochondrial function and decreased the ATP content; oxidative phosphorylation complex I activity; and oxidative phosphorylation complexes I, III, and V protein levels [31]. In a similar study, chronic ozone exposure in mice increased the pulmonary protein levels of DRP1 and oxidative phosphorylation complexes II and IV [30]. Lastly, in an acute exposure study in mice, ozone exposure increased the abundance of MFN2 and oxidative phosphorylation complexes II and IV [32]. Discrepancies between these studies and our study regarding the effect of ozone on mitochondrial markers may possibly be due to differences in the exposure model (chronic vs. acute) or differences in ozone concentrations (≥2.5 ppm in other studies vs. 0.8 ppm in our study). In addition, the previously mentioned studies assessed the outcome parameters 24 h after exposure, whereas we measured them within two hours after exposure, which may not have been enough time for the molecular responses to be detectable.

Although the perinatal HFD influenced the molecular responses to ozone, the perinatal HFD on its own (i.e., without the additional ozone exposure) did not have significant effects on the pulmonary oxidant status or molecular regulation of the mitochondrial content. However, maternal obesity has been demonstrated to have negative effects on oxidative stress and mitochondria in other tissues. Saben et al. found that maternal diet-induced metabolic syndrome increased lipid peroxidation and impaired mitochondrial morphology, dynamics, and metabolism in offspring muscle tissue and oocytes [39]. Similarly, Bruce et al. reported a perinatal HFD increased the expression of genes related to oxidative stress and decreased the activity of all electron transport chain complexes in offspring livers [40]. Lastly, in a study by Larsen et al., a prenatal HFD altered the mitochondrial morphology and impaired mitochondrial dynamism in offspring primary cardiomyocytes, with reductions in both fusion and fission events [41]. It should be noted, however, that none of these studies assessed their outcomes in lung tissue, and we were not able find any studies that did, which may partly explain the difference in findings between our and previous studies.

In the current study, the offspring animals were exposed to ozone concentrations of 0.8 ppm, which is higher than the ozone levels in controlled human intervention studies. It should be considered, however, that animals were exposed during the day, when rats are physically inactive. Humans, in contrast, either during real-life outdoor exposure or in controlled clinical studies, are generally exposed to ozone during physically active periods. Because of this difference, an ozone exposure of 0.8–1.0 ppm in rats is comparable to an exposure of 0.2–0.4 ppm in humans [42].

The ozone exposure in this study took place when the offspring animals were at an age of forty days, which is comparable to a peri-adolescent age in humans. Around this age of forty days, gonadal hormone concentrations rise in rat, with increases of progesterone and estradiol in females and testosterone in males [43]. As these sex hormones have been associated with ozone susceptibility, the sex differences observed in current study may have been different if the offspring animals would have been exposed and assessed at a different age. Nevertheless, exposing rats at an age of forty days is highly relevant, as children at this peri-adolescent age spend a relatively large part of their time outdoors, resulting in the highest real-life ozone exposure. In addition, children and teens, compared with other populations, are especially vulnerable to the effects of breathing ozone [44].

In contrast to what we hypothesized and found in the literature, many of the molecular markers that we measured in this study were not affected by ozone exposure. As already mentioned in this discussion, this may partly be due to the short amount of time between ozone exposure and tissue collection (two hours), potentially providing an insufficient time for molecular responses to develop. It is, in fact, known that although ozone exposure induces lung injury and inflammation immediately after exposure, the peak in these responses is observed the next day [45,46]. It may thus be better for future studies to assess molecular outcomes the day after exposure. Another limitation of our study is that mitochondrial outcomes were assessed only on the level of mRNA and protein expression. Although these outcomes provide valuable insights into the molecular responses to perinatal HFD and ozone exposure, it remains unclear whether they represent changes in mitochondrial function as well. Future studies may address this limitation by additionally assessing functional mitochondrial outcomes, such as the mitochondrial copy number, oxidative phosphorylation enzyme activity, and live imaging of fusion and fission events. In addition, future studies may also expand their focus to other relevant organs, such as the liver, given the involvement of oxidative stress and mitochondria in various liver conditions [47,48], and the adverse effects both perinatal HFD and ozone exposure are known to have on the liver [40,49,50]. Next to this, future studies may use a chronic exposure model instead of the acute exposure used in current study. Combining a perinatal HFD with such chronic ozone exposure may very well lead to different results than those reported in our study, as previous research has shown that repeated ozone exposure over several days leads to the attenuation of pulmonary responses [12,51,52].

In summary, we showed that acute ozone exposure increased pulmonary oxidant status and upregulated the molecular control of mitophagy, and that in female offspring these responses were exacerbated by a perinatal HFD. Considering the current obesity pandemic and a substantial part of the urban population being exposed to harmful ozone levels, investments should be made into improving early life nutrition and reducing exposure to air pollution to protect society against adverse health effects.

## 4. Materials and Methods

### 4.1. Animals, Diet, and Breeding

**Figure 7:**
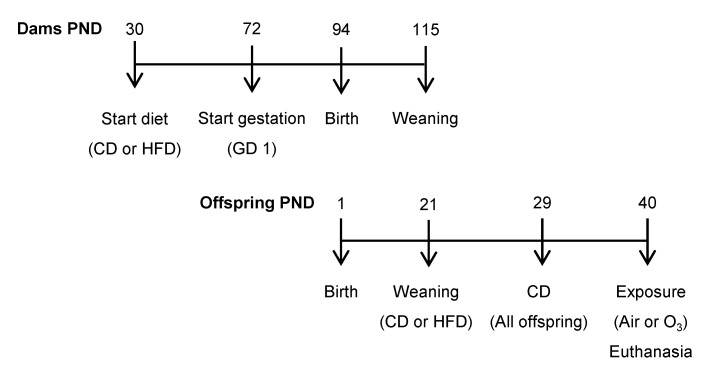
Experimental design. PND—postnatal day; GD—gestational day; CD—control diet; HFD—high-fat diet; O_3_—ozone.

The animal experiments were approved by the U.S. Environmental Protection Agency’s (EPA) Institutional Animal Care and Use Committee (LAPR#18-06-002). All of the animals were SPF-free, housed under 12 h/12 h light/dark cycles, and had ad libitum access to food and water. Initial feeding and breeding procedures were performed by Charles River Laboratories (Raleigh, NC, USA). Thirty-day-old female Long-Evans rats were fed either a control diet (CD; 10 kcal% fat; TD.08806, Harlan Laboratories, Teklad Custom Research Diets, Madison, WI, USA) or a high-fat diet (HFD; 60 kcal% fat, TD.06414) throughout the experiment (Figure 7). Females were bred on postnatal day (PND) 72 and shipped to the close-by EPA animal facility on gestational day (GD) 1. From each litter (*n* = 10 per diet group), two pups per sex were randomly selected and allocated to either the control or ozone exposure group (1 pup/sex/litter/exposure; *n* = 10 pups per sex/exposure/diet group). The offspring were first weaned onto the maternal diet (CD or HFD) on PND 21, and switched to CD on PND 29.

### 4.2. Ozone Exposure

On PND 40, the offspring were exposed to either filtered air or ozone (0.8 ppm) for 5 h. The animals were exposed in Rochester style “Hinners” chambers. Ozone was generated from oxygen using a silent arc discharge generator (OREC, Phoenix, AZ, USA) and was transported to the exposure chambers. All of the exposures occurred between 7 am and 11 am so as to avoid diurnal variation in biological endpoints. The mean (± standard error of the mean (SEM)) air temperature, relative humidity, and ozone concentration in the exposure chambers were 22.47 ± 0.14 °C, 52.72 ± 0.22%, and 0.0 ± 0.0 ppm, respectively, for filtered air exposure, and 23.24 ± 0.12 °C, 51.09 ± 0.22%, and 0.7999 ± 0.0016 ppm, respectively, for ozone exposure.

### 4.3. Euthanasia and Sample Collection

Within two hours after filtered air or ozone exposure, the animals were euthanized with an overdose of sodium pentobarbital (>200 mg/kg; Fatal Plus, Vortech Pharmaceuticals, LTD., Dearborn, MI, USA). The unlavaged left lung was snap frozen and stored at −80 °C until further assessment of the glutathione levels, total antioxidant capacity, lipid peroxidation, gene expression, and protein expression.

### 4.4. Glutathione Status

The glutathione status was assessed by measuring reduced and oxidized glutathione (GSH and GSSG, respectively). To measure GSSG, the samples were first depleted of GSH by incubating the lung homogenates with 3% (*v*/*v*) 2-vinylpyridine (Sigma-Aldrich, Saint Louis, MO, USA) and 6% (*v*/*v*) ethanol for 1 h at room temperature. To measure the GSH + GSSG, lung homogenates were prepared in 1.3% (*w*/*v*) 5-sulfosalicylic acid (Sigma-Aldrich) so as to prevent the oxidation of GSH. Reactions were started by adding 100 µL reagent, containing 0.3 mM 5,5-dithiobis(2-nitro-benzoic acid) (Sigma-Aldrich) and 0.4 mM NADPH (Sigma-Alrich), and 50 µL glutathione reductase (4 U/mL) to 50 µL prepared lung homogenates. Absorbance was recorded at 412 nm for 3 min at 20 s intervals. GSSG and GSH + GSSG concentrations were determined using the reaction rate and standard curves. The GSH and GSSG concentrations were normalized to the starting amount of lung tissue. The GSSG/GSH ratio was calculated as a measure for the glutathione status.

### 4.5. Total Antioxidant Capacity

The total antioxidant capacity was determined using the trolox equivalent antioxidant capacity (TEAC) assay. Lung homogenates were deproteinized by incubating samples in 5% trichloric acid for 5 min on ice, followed by centrifugation at 15,000 rpm for 5 min. To assess the antioxidant capacity, supernatants were incubated with a radical solution, containing 5 mM 2,2 azino-bis-3 ethylbenzothiazoline-6-sulfonic acid (Sigma-Aldrich), 20 µM H_2_O_2_, and 20 mU/mL horseradish peroxidase (Sigma-Aldrich) at 37 °C for 5 min, and the absorbance was measured at 734 nm. TEAC was calculated by normalizing the absorbances to an antioxidant capacity of 1 µM Trolox, and was further normalized to the starting amount of lung tissue.

### 4.6. Lipid Peroxidation

The lipid peroxidation was assessed by measuring the malondialdehyde (MDA) levels using the thiobarbituric acid reactive substances (TBARS) assay. Lung homogenates were incubated with a reagent containing 12 mM 2-thiobarbituric acid (Merck-Millipore, Burlington, MA, USA), 0.01% (*w*/*v*) EDTA (Merck-Millipore), 320 mM H_3_PO_4_, and 6.81 mM butylated hydroxytoluene (Sigma-Aldrich) at 100 °C for 1 h. The reaction was stopped by putting samples on ice, 0.5 vol. 1-butanol was added, and the samples were vortexed and centrifuged at 12,000 rpm for 3 min. Fluorescence was measured in supernatant at λ_exc._ = 530 nm and λ_em._ = 560 nm. The MDA levels were quantified using a standard curve and were normalized to the starting amount of lung tissue.

### 4.7. Gene Expression

The total RNA was extracted from the offspring lung using TRIzol Reagent (Thermo Fischer Scientific, Waltham, MA, USA). Then, 400 ng RNA was reverse transcribed into the cDNA with iScript (Bio-Rad, Hercules, CA, USA). Real-time qPCR was performed with 4.4 µL of 1:50 diluted cDNA, target-specific primers (Table S2), SensiMix SYBR and Fluorescein Kit (Meridian Bioscience, Cincinnati, OH, USA), and a LightCycler 480 (Roche, Basel, Switzerland). The gene expression levels were quantified with LinRegPCR software version 2014.0 [53] and normalized using the geometric mean of the expression levels of the reference genes *Tuba1b*, *Rpl13a*, and *Ppia*. Samples with non-specific melt curves or sub-optimal amplification (detected as unstable baseline or no plateau in the amplification curve by LinRegPCR software) were excluded from the analysis, leading to *n* = 9–10 per group for all of the examined genes.

### 4.8. Protein Expression

The offspring lung lysates were prepared in an immunoprecipitation buffer (Table S3). The protein concentrations of the lysates were measured with Pierce BCA Protein Assay Kit (Thermo Fischer Scientific, Waltham, MA, USA). Lysates were boiled in a 1× Laemmli buffer (Table S4) and 10 µg protein per sample was separated on 12% Criterion XT Bis-Tris Protein Gel (Bio-Rad) and blotted onto 0.45 µm nitrocellulose membranes (Bio-Rad). The membranes were stained for the total protein with PonceauS (Sigma-Aldrich, Saint Louis, MO, USA) and imaged on the Amersham Imager 600 (GE Healthcare, Chicago, IL, USA). For the antigen-specific expression levels, the membranes were probed with primary antibodies (Table S5) and HRP-conjugated secondary antibodies (Table S6), incubated with SuperSignal West PICO or Femto Chemiluminescent Substrate (Thermo Fisher Scientific), and imaged on the Amersham Imager 600. The total protein levels and antigen-specific expression were quantified with ImageJ version 1.53e [54]. The antigen-specific expression was normalized for the total protein levels. Given the large number of animals, the samples were analyzed on four different gels/blots. To prevent bias from this, an equal number of samples from each experimental group was analyzed on each gel/blot. In addition, between-gel/blot variation was removed using factor correction [55], which was confirmed using principle component analysis. For some offspring animals, insufficient tissue was left over for the protein analysis after all of the previous measurements, leading to *n* = 7 for ozone-exposed CD females and *n* = 9–10 for all of the other experimental groups.

### 4.9. Statistical Analysis

All data were stratified for sex and analyzed using two-way ANOVA, with diet and exposure as factors. First, it was checked whether both factors had a significant interaction. If not, the interaction term was removed from the analysis. Next, it was tested whether one or both factors had a significant effect on the outcome. For those factors with a significant effect, Holm–Sidak post-hoc test was used to compare the means. In addition to these primary analyses, associations were assessed between the glutathione status, total antioxidant capacity, lipid peroxidation, gene expression, and protein expression vs. previously published outcomes from the same experiment, such as markers for lung injury and inflammation [19]. These associations were determined using Pearson’s product–moment correlation after stratification for sex and diet. Given the large number of correlations that were tested, *p*-values were corrected for multiple testing by computing the false discovery rate q-values using the Benjamini–Hochberg procedure [56]. The results were graphically presented as boxplots, with boxes representing the first quartile, median, and third quartile, and whiskers representing the minimum and maximum values within 1.5 times the interquartile range (IQR) below the first quartile or above the third quartile, respectively, and with points representing the values outside the limits of the whiskers. *p* < 0.05 or q < 0.05 was considered significant. All of the statistical analyses were performed in R version 4.0.2 (R Foundation for Statistical Computing, Vienna, Austria).

## Figures and Tables

**Table 1:** Lung *Sod2* mRNA levels correlated significantly with broncheoalveolar lavage fluid (BALF) vascular leakage markers albumin and total protein in female and male offspring from high-fat diet (HFD) dams. All of the markers were assessed within two hours after exposure to filtered air or ozone in the lungs or BALF from female and male offspring from dams fed a perinatal control diet (CD) or HFD. BALF albumin and total protein data were previously published [19]. Only significant correlations (q < 0.05) are shown in this table. The results from non-significant correlations are listed in Table S1. Statistical analysis by Pearson’s product–moment correlation after stratification for sex and diet. False discovery rate q-values were computed from *p*-values using the Benjamini–Hochberg procedure to correct for multiple testing.

Correlation	Stratum	r	q-Value
*Sod2* mRNA vs. BALF albumin	Female CD	0.42	0.624
	Female HFD	0.78	0.014
	Male CD	0.59	0.462
	Male HFD	0.82	0.003
*Sod2* mRNA vs. BALF total protein	Female CD	0.49	0.550
	Female HFD	0.77	0.014
	Male CD	0.58	0.462
	Male HFD	0.80	0.004

## Data Availability

Data presented in this study, including untouched Western blot images, are available from the corresponding author upon request.

## Supplementary Materials

The following are available online at https://www.mdpi.com/article/10.3390/ijms22147551/s1.
