# Supplementary material for: Perinatal High-Fat Diet Influences Ozone-Induced Responses on Pulmonary Oxidant Status and the Molecular Control of Mitophagy in Female Rat Offspring
Source: Int J Mol Sci. 2021 Jul 14;22(14):7551. doi: 10.3390/ijms22147551 (PMC8304403; doi:10.3390/ijms22147551)
Supplement: Supplementary file 1 [file ijms-22-07551-s001.zip › Rouschop_IJMS_2021_Supplemental materials.pdf]

Table S2. Primer sequences for real-time quantitative PCR.

Gene	Forward primer (5′-3′)	Reverse primer (5′-3′)
Reference genes		
Tubab1b	AGCGCAGCATCCAGTTTGT	CTGTGGTGTTGCTCAGCATAGA
Rpl13a	GCGGATGAACACCAACCCGT	CAGCCTGGCCTCTTTTGGTCT
Ppia	TCCATGGCAAATGCTGGACCAA	CCTGGACCCAAAACGCTCCA
Antioxidants		
Cat	GAATGGCTATGGCTCACACA	CAAGTTTTTGATGCCCTGGT
Sod1	CGAGCATGGGTTCCATGTC	CTGGACCGCCATGTTTCTTAG
Sod2	ATTAACGCGCAGATCATGCA	CCTCGGTGACGTTCAGATTGT
Mitophagy		
Pink1	CCAAACACCTTGGCCTTCTA	CTTAAGATGGCTTCGCTGGA
Bnip3	CAGAGCGGCGAGGAGAACCTGCAG	GCTGCTCCCATTCCCATTGCTGAAG
Bnip3l	AGGCTAACCTGCAGCACAGT	CACTGCCGATGAAACTGCTA
Mitochondrial biogenesis		
Ppargc1a	GGGACATGTGCAGCCAAGACT	GATCTGGGCAAAGAGGCTGGT
Ppargc1b	AGCGCTTCGAGGTGTTTGGT	TTCTCAGGGTAGCGCCGTTC
Tfam	AATTGCAGCCATGTGGAGGGAG	GCCGGGCTTCCTTCTCTAAGC
Mitochondrial fusion		
Mfn1	CGGAGGCATATGAAAGTGGC	CCATCAGTTCCCTCCACACT
Mfn2	TTGACTCCAGCCATGTCCAT	GGTGACGATGGAGTTGCATC
Opa1	CAGCTGGCAGAAGATCTCAAG	CATGAGCAGGATTTTGACACC
Mitochondrial fission		
Drp1	TGGAGATGGTGGTCAGGAAC	TTTCGTGCAACTGGAACTGG
Fis1	ATGGATGCCCAGAGATGAAG	ACGATGCCTCTACGGATGTC
Oxidative phosphorylation		
Ndufb3	GAAGAAGCTTGCTGCACGAGG	ACGCAGCAAACCCCCATTTG
Sdhb	GAACGGAGACAAGTACCTGGGG	GATGGTGTGGCAGCGGTAGA
Cyc1	ATGTTGCCACCTTCCTTCGCT	AGGACTGACCACTTATGCCGC
Cox4i1	CTGAAGGAGAAGGAGAAGG	CAGTGAAGCCGATGAAGA

Table S3. Immunoprecipitation buffer composition

Component	Concentration
Tris	50 mM
NaCl	150 mM
Glycerol	10% (w/v)
NP-40	0.05% (w/v)
Ethylenediaminetetraacetic acid (EDTA)	1 mM
cOmplete protease inhibitor cocktail*	1x
PhosSTOP phosphatase inhibitor*	1x
pH	7.4

*Roche, Basel, Switzerland

Table S4. Laemmli buffer (4x)

Component	Concentration
Tris	0.25 M
Sodium dodecyl sulfate (SDS)	8% (w/v)
Glycerol	40% (w/v)
Dithiothreitol (DTT)	0.4 M
Bromophenol Blue	0.02% (w/v)
pH	6.8

Table S5. Western blot primary antibodies

Antigen	Dilution	Supplier
BNIP3	1:1000	Cell Signaling Technology
BNIP3L	1:1000	Cell Signaling Technology
SOD2	1:1000	Santa Cruz Biotechnology
OXPHOS I-V	1:1000	Abcam

Table S6. Western blot secondary antibodies

Primary antibody	Secondary antibody	Dilution	Supplier
BNIP3	Anti-rabbit	1:10,000	Vector Laboratories
BNIP3L	Anti-rabbit	1:10,000	Vector Laboratories
SOD2	Anti-mouse	1:10,000	Vector Laboratories
OXPHOS I-V	Anti-mouse	1:10,000	Vector Laboratories
